# Serum uric acid to creatinine ratio and risk of preeclampsia and adverse pregnancy outcomes

**DOI:** 10.1097/HJH.0000000000003472

**Published:** 2023-06-05

**Authors:** Federica Piani, Davide Agnoletti, Alessandro Baracchi, Sara Scarduelli, Carmine Verde, Giovanni Tossetta, Elisa Montaguti, Giuliana Simonazzi, Daniela Degli Esposti, Claudio Borghi

**Affiliations:** a Cardiovascular Internal Medicine Unit, IRCCS Azienda Ospedaliero-Universitaria di Bologna; b University of Bologna, Department of Medical and Surgical Sciences; c Obstetrics and Gynecology Unit, IRCCS Azienda Ospedaliero-Universitaria di Bologna, Bologna; d Department of Experimental and Clinical Medicine, Polytechnic University of Marche, Ancona, Italy

**Keywords:** hypertension, preeclampsia, pregnancy, pregnancy complications, pregnancy-induced hypertension, prevention and control, SUA/sCr, uric acid, uric acid creatinine ratio

## Abstract

Preeclampsia is one of the most severe diseases among the hypertensive disorders of pregnancy (HDP) and the leading cause of maternal and fetal morbidity and mortality. It is of crucial importance to early identify women at a high risk for preeclampsia to implement appropriate preventive strategies. In our study, we aimed to test the hypothesis that serum uric acid to creatinine ratio (SUA/sCr) is related to the development of preeclampsia and maternal and neonatal complications.

**Methods:**

We searched for uric acid and creatine values in the medical records of 269 women who consecutively attended our HDP Clinic from December 2018 to December 2022. We compared the baseline characteristics of participants with normotensive pregnancy (*n* = 57), to those with HDP without preeclampsia (HDP-non-PE) (*n* = 100) and those with preeclampsia (*n* = 112), and we performed adjusted logistic regression analysis to test the associations between SUA/sCr and the development of preeclampsia and maternal and neonatal complications.

**Results:**

SUA/sCr was consistently higher in women with preeclampsia in all trimesters of pregnancy. Higher SUA/sCr at the third trimester was associated with an increased odd of developing preeclampsia [odds ratio (OR) 1.29, confidence interval (CI) 1.15–1.50, *P* = 0.001], preterm birth (OR 1.23, CI 1.05–1.45, *P* = 0.011), and composite neonatal outcome (OR 1.33, CI 1.12–1.59, *P* = 0.001), after adjustment for age, BMI before pregnancy, nulliparity, antihypertensive therapy, and acetylsalicylic acid therapy during pregnancy.

**Conclusions:**

Having higher SUA/sCr during pregnancy is associated with the development of PE and adverse pregnancy outcomes. Controlled prospective studies are warranted to clarify the predictive power of this novel marker during pregnancy.

## INTRODUCTION

Preeclampsia is a hypertensive disorder of pregnancy (HDP) characterized by trophoblast immaturity and vascular dysfunction [1,2], and represents one of the major causes of maternal and fetal morbidity and mortality [3]. Although preeclampsia incidence is increasing worldwide, the underlying pathophysiological mechanisms remain poorly understood with consequent delays on the development and availability of novel diagnostic tests [4]. Currently, the Fetal Medicine Foundation algorithm for the first-trimester prediction of preeclampsia can predict around 41% of term-preeclampsia (≥37 weeks of pregnancy), and 70–75% of preterm preeclampsia (<37 weeks) [4]. As preterm and term preeclampsia account for more than 50% of total preeclampsia cases, our predictive capacity for preeclampsia might be far less than 70%. For this reason, there is a need for efficient and widely available markers able to increase the predictive capacity for preterm preeclampsia and preeclampsia-associated maternal and neonatal complications.

Serum uric acid, the end-product of purine catabolism, has been associated with the development of preeclampsia since 1925 [5]. From that report, several studies confirmed the predictive value of serum uric acid for preeclampsia and preeclampsia-associated complications [6]. As hyperuricemia is a complex entity that can be caused by very diverse conditions such as kidney injury (decreased uric acid excretion) and hyperactivity of xanthine oxidase (hyperproduction of uric acid), serum uric acid to creatinine ratio (SUA/sCr) has recently been proposed as a better marker than uric acid alone [7]. In fact, SUA/sCr may be able to differentiate among subgroups of hyperuricemic patients. This is particularly important because hyperuricemia due to kidney injury may be less associated with the development and progression of cardiovascular diseases [8]. Interestingly, accumulating evidence suggests that HDP, in particular preeclampsia, are maternal vascular disorders characterized by endothelial dysfunction and angiogenic imbalance with a life-long increased cardiovascular risk [9,10]. Hyperuricemia due to hyperactivity of xanthine oxidase produces reactive oxygen species and is associated with the development of endothelial and vascular dysfunction [2,11]. This pathogenetic mechanism may play a crucial role in the development and severity of preeclampsia [12]. Thus, the importance of considering uric acid values adjusted for serum creatinine, to identify hyperuricemic women who may be at a higher risk of the well known long-term cardiovascular complications associated to HDP and preeclampsia. In the present study, we aim to assess the trends of SUA/sCr at each trimester of pregnancy and to investigate the associations between SUA/sCr and the development of preeclampsia and adverse maternal and neonatal outcomes in a cohort of women with HDP and normotensive pregnancies.

## MATERIALS AND METHODS

### Study participants and data collection

The present study is a combined retrospective and prospective study carried out in IRCCS Policlinico Sant’Orsola-Malpighi, Bologna, Italy. Pregnant women who attended the HDP Clinic of our Cardiovascular Internal Medicine Unit from December 2018 to December 2022 were enrolled in this study. Women were referred to our Clinic by the Obstetrics and Prenatal Medicine Department if they were considered at risk of developing HDP or after they developed HDP. Thus, in our study, a high proportion of women was diagnosed with HDP-non-preeclampsia and preeclampsia, compared with those who had normotensive pregnancies. The study participants were divided into three groups: 100 women with HDP-non-preeclampsia, 112 women with preeclampsia, and 57 women who had normotensive pregnancies but were considered at moderate-high risk of developing HDP and preeclampsia at the beginning of their pregnancy. The different groups of participants have not been matched. HDP and preeclampsia were defined according to the International Society for the Study of Hypertension in Pregnancy (ISSHP). Exclusion criteria were the absence of at least one concomitant measurement of serum uric acid and creatinine throughout the pregnancy or after giving birth, and the presence of more than one fetus. Values of serum creatinine and uric acid have been grouped into four timepoints: first trimester (1–13 weeks), second trimester (14–26 weeks), third trimester (27–40 weeks), and postpartum (from 48 h after delivery to 1 week after delivery).

Women were classified as acetylsalicylic acid (ASA) users if they were prescribed with ASA before 16th weeks of pregnancy and continued the therapy until 35 weeks of pregnancy. Women were considered under antihypertensive therapy if they needed any class of antihypertensive drug during pregnancy and for at least 1 week. All the cases were followed until the delivery for maternal and neonatal outcomes, namely, neonatal or maternal death, need for ICU or neonatal ICU (NICU), preterm birth (before 37 weeks of pregnancy), low weight at birth (<2500 g), development of gestational diabetes during pregnancy [the diagnosis was made if any of the following glucose thresholds were met during a fasting 75 g 2-h oral glucose tolerance test (OGTT): fasting ≥92 mg/dl; 1 h ≥180 mg/dl; 2 h ≥153 mg/dl, [13]].

Serum uric acid and creatinine values (mg/dl) used in the study were measured in the Laboratory of Bologna Metropolitan Area (LUM), an accredited public laboratory following European standard for quality requirements in medical laboratories. Serum creatinine and serum uric acid were determined by spectrophotometry: Jaffe's reaction and uricase-peroxidase system were used, respectively.

A written informed consent was obtained from women agreeing to participate in the study. All participants were treated according to Helsinki declaration of biomedical ethics. The institutional ethics committee (CE AVEC) approved the study. All participants underwent standard clinical care and attended regular scheduled visits to their obstetricians and midwives and to our HDP Clinic, according to Hospital protocols and counseling from their healthcare provider.

### Statistical analysis

Continuous variables are presented as mean ± standard deviation (SD) or as median and interquartile range (IQR) based on distribution and skewness, while categorical variables are expressed as numbers and percentages. For the comparisons between groups, one-way analysis of variance associated with a post hoc test (Bonferroni) and the Kruskal--Wallis H test with subgroups posthoc analysis were used for parametric and nonparametric variables, respectively. To assess the associations between SUA/sCr and maternal and neonatal outcomes, odds ratios (ORs) with 95% confidence intervals (95% CIs) were obtained by adjusted logistic regression models. ORs were adjusted for age, BMI before conception, nulliparity, antihypertensive therapy during pregnancy, acetylsalicylic acid therapy during pregnancy. All statistical tests were two-tailed, and *P* values less than 0.05 were considered statistically significant. The analyses were conducted using the Statistical Package for the Social Science (SPSS) version 28 [SPSS Inc., Chicago, Illinois, USA], Macintosh Version.

## RESULTS

### Participants’ characteristics by group (normotensive pregnancies vs. HDP-non-preeclampsia vs. preeclampsia)

Mean age of the participants did not differ among groups. BMI was significantly higher in women with HDP than in women with normotensive pregnancies (*P* = 0.009), corroborating existing literature on the association between BMI and HDP development in Italian and International cohorts of pregnant women [14,15]. Nulliparity rate was significantly higher in women with preeclampsia vs. other groups (*P* = 0.018). The trend remained consistent when considering the number of the current pregnancy, with women affected by preeclampsia showing lower number of previous pregnancies compared with women with HDP-non-preeclampsia and normotensive controls (*P* = 0.014). The need for antihypertensive therapy was similar in HDP-non-preeclampsia and preeclampsia groups, and the rate of participants under ASA was lower in preeclampsia group (*P* = 0.008). Women with HDP-non-preeclampsia compared with those who developed preeclampsia and those with normotensive pregnancies had a significantly higher BMI (27.7 vs. 25.9 vs. 25.4 kg/m^2^, respectively; *P* = 0.009) and had more frequently four or more previous pregnancies (19 vs. 7–8% in the other groups; *P* = 0.014). Forty percent of women in the HDP-non-preeclampsia group were prescribed with acetylsalicylic acid during pregnancy vs. only 21.4% in the preeclampsia group and 28.1% in the normotensive pregnancies group (*P* = 0.008). This may partially explain why we did not observe significant differences in maternal and neonatal outcomes between women with HDP-non-preeclampsia and women with normotensive pregnancies. The incidence of maternal and neonatal adverse outcomes was higher in preeclampsia group than in both HDP-non-preeclampsia and normotensive pregnancies. Six out of seven women who needed to be transferred to the ICU were in the preeclampsia group. Women with preeclampsia had a significantly higher proportion of babies with low body weight at birth compared with both HDP-non-preeclampsia and normotensive pregnancies (51.8 vs. 15 vs. 10.5%, respectively; *P* < 0.001). Likewise, newborns from mothers with preeclampsia more frequently needed to be transferred to the NICU (25.9% vs. about 5% in both other groups; *P* < 0.001). Neonatal death occurred in four of the 269 babies, and only in the preeclampsia group (Table 1).

**TABLE 1:** General characteristics of the study groups.

	NP (*n* = 57)	HDP-non-PE (*n* = 100)	PE (*n* = 112)	*P* value
Age (years)	35 ± 5	36 ± 5	35 ± 6	0.236
BMI (kg/m^2^)	25.4 (6.4)	27.7 (9.3)^a^	25.9 (7.2)^b^	**0.009**
Spontaneous pregnancy (*n*, %)	53 (93%)	90 (90%)	102 (91.1%)	0.952
Nulliparity (*n*, %)	22 (38.6%)	37 (37%)	60 (53.6%)^a^^,^^b^	**0.018**
Number of current pregnancy:				**0.014**
Second	19/57 (33.3%)	33/100 (33%)	25/112 (22.3%)^a^^,^^b^	
Third	8/57 (8.8%)	11/100 (11%)	12/112 (10.7%)	
≥Fourth	8/57 (8.8%)	19/100 (19%)^a^	8/112 (7.1%)	
Gestational diabetes (*n*, %)	13 (22.8%)	23 (23%)	29 (25.9%)	0.981
Antihypertensive therapy (*n*, %)	0 (0%)	80 (80%)	92 (82.1%)	n.a.
Acetylsalicylic acid (*n*, %)	16 (28.1%)	40 (40%)	24 (21.4%)^b^	**0.008**
Spontaneous delivery (*n*, %)	32 (56.1%)	44 (44%)	35 (31.3%)^a^	**0.035**
Preterm delivery (*n*, %)	3 (5.3%)	7 (7%)	48 (42.9%)^a^^,^^b^	**<0.001**
Low neonatal body weight (*n*, %)	6 (10.5%)	15 (15%)	58 (51.8%)^a^^,^^b^	**<0.001**
Need for NICU (*n*, %)	3 (5.3%)	5 (5%)	29 (25.9%)^a^^,^^b^	**<0.001**
Need for ICU (*n*, %)	1 (1.7%)	0 (0%)	6 (5.4%)^b^	n.a.
Neonatal death (*n*, %)	0 (0%)	0 (0%)	4 (3.6%)	n.a.
Maternal death (*n*, %)	0 (0%)	0 (0%)	0 (0%)	n.a.

*P* values in bold indicate statistical significance for the ANOVA among groups. HDP, hypertensive disorders of pregnancy; NICU, neonatal ICU; NP, normotensive pregnancies; PE, preeclampsia; SUA/sCr, serum uric acid to creatinine ratio.

a Significant difference compared with NP.

b Significant difference compared with HDP-non-PE.

### Serum uric acid to creatinine ratio comparisons among groups (normotensive pregnancies vs. HDP-non-preeclampsia vs. preeclampsia)

**FIGURE 1:**
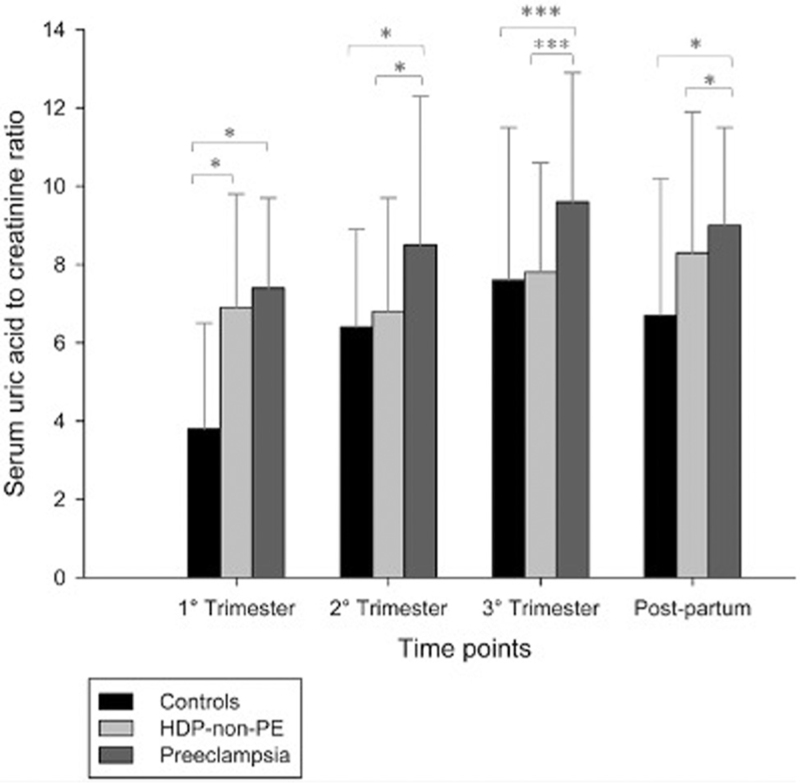
The distribution of serum uric acid to creatinine ratio by study group and study timepoints. HDP-non-PE, hypertensive disorders of pregnancy without preeclampsia; PE, preeclampsia. Asterisks indicate statistical significance of posthoc comparisons among groups: ^∗^*P* < 0.05, ^∗∗^*P* < 0.005, ^∗∗∗^*P* < 0.001.

Only 12 patients had data on SUA/sCr at all timepoints (two controls, four HDP, six preeclampsia). Most patients had only one measurement of SUA/sCr (*n* = 208), while 61 patients had at least two measurements (nine controls, 28 HDP, 24 preeclampsia). SUA/sCr was significantly higher in women with preeclampsia than in women with HDP-non-preeclampsia and normotensive pregnancies throughout all timepoints (*P* = 0.005, 0.030, <0.001, 0.008, respectively). In all timepoints except for the first trimester, SUA/sCr was significantly different between normotensive pregnancies and preeclampsia, and between preeclampsia and HDP-non-preeclampsia (Fig. 1; Table 2).

**TABLE 2:** Serum uric acid and creatinine values by study group and study timepoints.

	NP First trimester: *n* = 8 Second trimester: *n* = 12 Third trimester: *n* = 26 Postpartum: *n* = 10	HDP-non-PE First trimester: *n* = 10 Second trimester: *n* = 30 Third trimester: *n* = 75 Postpartum: *n* = 46	PE First trimester: *n* = 10 Second trimester: *n* = 27 Third trimester: *n* = 85 Postpartum: *n* = 80	*P*
Serum uric acid (mg/dl) first trimester	2.4 (1.8)	4.6 (2.5)^a^	3.7 (1.3)	**0.043∗**
Serum creatinine (mg/dl) first trimester	0.63 (0.02)	0.57 (0.19)	0.52 (0.17)^a^^,^^b^	**0.037∗**
Serum uric acid (mg/dl) second trimester	3.5 (1.3)	3.5 (1.2)	4.1 (2.5)	0.077
Serum creatinine (mg/dl) second trimester	0.52 (0.05)	0.51 (0.10)	0.56 (0.18)	0.563
Serum uric acid (mg/dl) third trimester	4.2 (1.5)	4.7 (2.1)	6.4 (2.3)	**<0.001∗∗∗**
Serum creatinine (mg/dl) third trimester	0.56 ± 0.12	0.60 ± 0.14	0.65 ± 0.15	**0.007∗**
Serum uric acid (mg/dl) postpartum	4.6 (1.6)	5.4 (2.2)	5.9 (1.8)	**0.002∗∗**
Serum creatinine (mg/dl) postpartum	0.63 ± 0.12	0.65 ± 0.13	0.65 ± 0.11	0.810

HDP, hypertensive disorders of pregnancy; NP, normotensive pregnancies; PE, preeclampsia; SUA/sCr, serum uric acid to creatinine ratio. *P* values in bold indicate statistical significance for the ANOVA among groups.

a Significant difference compared with NP.

b Significant difference compared with HDP. ^∗^*P* < 0.05, ^∗∗^*P* < 0.005, ^∗∗∗^*P* < 0.001.

### Associations between serum uric acid to creatinine ratio, preeclampsia development, and maternal and neonatal outcomes

SUA/sCr values at postpartum were not considered for this analysis, as outcomes had already occurred. Higher SUA/sCr at the third trimester was significantly associated with an increased odd for PE development (OR 1.32, CI 1.14–1.53, *P* < 0.001), while SUA/sCr at other timepoints was not associated with preeclampsia development. Considering a composite maternal outcome including the need for ICU and preterm delivery, higher SUA/sCr at third trimester was associated with an increased odd for maternal events (OR 1.31, CI 1.11–1.55, *P* = 0.002). Higher SUA/sCr at third trimester was associated with an increased odd for a composite neonatal outcome (OR 1.19, CI 1.04–1.37, *P* = 0.012), including low weight at birth, need for NICU, and neonatal death (Table 3). SUA/sCr at first and second trimester of pregnancy was not associated with a significant change in occurrence of maternal or neonatal outcomes.

**TABLE 3:** Logistic regression models of the association between serum uric acid to creatinine ratio at third trimester of pregnancy and the development of adverse maternal and neonatal outcomes.

	O.R.	S.E.	Exp(B) with 95% CI	*P* value
Development of PE				
SUA/sCr third trimester	1.321	0.076	1.139–1.533	**<0.001** ^**∗∗∗**^
Age (years)	0.985	0.037	0.917–1.059	0.686
BMI before pregnancy (kg/m^2^)	0.942	0.028	0.891–0.996	**0.037** ^**∗**^
Nulliparity (yes/no)	1.399	0.380	0.665–2.943	0.377
Antihypertensive therapy (yes/no)	5.285	0.452	2.181–12.806	**<0.001** ^**∗∗∗**^
ASA therapy during pregnancy (yes/no)	0.283	0.410	0.127–0.633	**0.002** ^**∗∗**^
Composite maternal outcome (need for ICU, preterm delivery)				
SUA/sCr third trimester	1.311	0.087	1.106–1.554	**0.002** ^**∗∗**^
Age (years)	0.995	0.039	0.922–1.074	0.896
BMI before pregnancy (kg/m^2^)	0.914	0.040	0.845–0.988	**0.024** ^**∗**^
Nulliparity (yes/no)	1.006	0.443	0.442–2.397	0.989
Antihypertensive therapy (yes/no)	3.291	0.658	0.906–11.955	0.070
ASA therapy during pregnancy (yes/no)	0.434	0.530	0.153–1.227	0.116
Composite neonatal outcome (low weight at birth, need for NICU, death)				
SUA/sCr third trimester	1.195	0.071	1.040–1.373	**0.012** ^**∗**^
Age (years)	0.976	0.036	0.911–1.047	0.502
BMI before pregnancy (kg/m^2^)	0.924	0.032	0.868–0.983	**0.013** ^**∗**^
Nulliparity (yes/no)	1.109	0.393	0.513–2.394	0.793
Antihypertensive therapy (yes/no)	8.750	0.659	2.404–31.846	**<0.001** ^**∗∗∗**^
ASA therapy during pregnancy (yes/no)	0.578	0.420	0.254–1.316	0.192

*P* values in bold indicate statistical significance. ASA, acetylsalicylic acid; CI, confidence interval; O.R., odds ratio; PE, preeclampsia; S.E., standard error; SUA/sCr, serum uric acid to creatinine ratio. ^∗^*P* < 0.05, ^∗∗^*P* < 0.005, ^∗∗∗^*P* < 0.001.

## DISCUSSION

In our study, for the first time, we investigated SUA/sCr values throughout all trimesters of pregnancy and postpartum in a cohort of women with HDP-non-preeclampsia, preeclampsia, and normotensive pregnancies. Furthermore, we assessed the associations of all trimesters SUA/sCr with adverse pregnancy outcomes. In our study, SUA/sCr values were significantly higher in women with preeclampsia during all timepoints compared with women with normotensive pregnancies and women with HDP non-preeclampsia. Notably, higher SUA/sCr levels measured at the third trimester of pregnancy were more likely associated to the development of preeclampsia and composite maternal (need for ICU, preterm delivery) and neonatal (low weight at birth, need for NICU, death) outcomes, even after adjustment for age, BMI before conception, nulliparity, antihypertensive therapy, and ASA therapy during pregnancy.

Physiologically, both serum uric acid and creatinine levels tend to decrease during the first and second trimester of pregnancy and to increase during the late second and third trimester of pregnancy compared to prepregnancy levels [16,17]. These changes are regulated by pregnancy hemodilution and increased vascular volume, changes in vascular resistances, regulation of renal filtration and secretion, increased concentration of hormones with vasoactive and diuretic effects, changes in albumin levels, and fetal growth and cellular metabolism levels [18–21]. Most of these factors are expression of an appropriate hemodynamic adaptation to pregnancy, a massive set of changes occurring in the maternal cardiovascular system that guarantee the correct fetal growth and development [22]. As HDP and preeclampsia are characterized by different degrees of hemodynamic maladaptation to pregnancy, SUA/sCr may represent an indirect and early risk marker of developing these conditions [23]. This hypothesis is corroborated by the evidence of xanthine oxidase hyperactivity in women with preeclampsia, suggesting that hyperuricemia associated with preeclampsia is caused by hyperproduction of uric acid rather than underexcretion secondary to renal function impairment [12].

The association between serum uric acid and preeclampsia has already been described more than 50 years ago [5], and serum uric acid measurement is recommended in the routine management of women with HDP by both European and American guidelines [3,24]. Serum uric acid has a strong association with arterial hypertension and cardiovascular disease [25–27]; however, studies on the predictive role of serum uric acid for preeclampsia and adverse pregnancy outcomes are not consistent nor strong enough to include it in the currently used risk scores for preeclampsia [6,28]. A possible explanation of result inconsistency is that hyperuricemia is not a single entity, but a condition associated with many causes. Thus, inappropriate stratification of patients with different causes of hyperuricemia may lead to selection biases and conflicting results. In fact, hyperuricemia due to increased xanthine oxidase activity may be the only form of hyperuricemia associated with the development of preeclampsia and its complications. For this reason, SUA/sCr rather than serum uric acid alone may increase our ability to predict preeclampsia development and adverse pregnancy outcomes, excluding hyperuricemia secondary to kidney disease [7].

To the best of our knowledge, only a recent retrospective case control study has investigated SUA/sCr during pregnancy, in women with preeclampsia and healthy controls (*n* = 84 and *n* = 86, respectively) [29]. The authors found that women with preeclampsia had higher SUA/sCr compared with healthy controls and that SUA/sCr positively associated with platelet-to-lymphocyte ratio of newborns. However, Yakiştiran *et al*. [29] did not compare the trends of SUA/sCr in all trimesters of pregnancy among women with HDP non-preeclampsia, women with preeclampsia, and women with normotensive pregnancies. Furthermore, they did not assess the associations between SUA/sCr during pregnancy and the odds of maternal and neonatal adverse outcomes. Other studies reported that both uric acid and creatinine serum levels increase in pregnancies complicated by preeclampsia and hold prognostic value for adverse pregnancy outcomes, but none of them calculated the combination of both biochemical indices, expressed in term of SUA/sCr [30–32]. As HDP prevalence is increasing worldwide and the only preventive measure for preeclampsia has shown to be the administration of ASA before the 16th week of pregnancy, it is of crucial importance to identify women at high risk for preeclampsia to early start treatment with ASA and plan an appropriate follow-up in a third referral center for HDP [33,34]. Both serum uric acid and serum creatinine are widely available biochemical analytes, and our study suggests that their ratio might improve the efficacy of current PE risk prediction. Furthermore, as SUA/sCr may help stratifying patients at higher cardiovascular risk, namely those with hyperuricemia due to increased uric acid production, SUA/sCr may represent a valid prognostic marker not only for adverse pregnancy outcomes but also for long-term cardiovascular outcomes of women with a previous history of HDP [35].

This study has important limitations worth mentioning, including the observational design, which does not allow to infer about causality, and limits the sample size of each subgroup of patients at each timepoint. Furthermore, timepoints are wide and we cannot exclude the influence of different weeks of gestations on the concentration of the studied analytes. For these reasons, our analyses should be considered hypothesis generating. Strengths of the study include the availability of data on every trimester of pregnancy, and the possibility to compare data not only between women with HDP and normotensive controls, but also between HDP-non-preeclampsia and preeclampsia. Large and longitudinal studies are warranted to better define the role of SUA/sCr in the pathogenesis and risk prediction of preeclampsia and the associated maternal and neonatal complications.

In conclusion, our study is the first to investigate the role of SUA/sCr in HDP pregnancies and its association with adverse pregnancy outcomes. We showed that women with preeclampsia had higher values of SUA/sCr compared with HDP-non-preeclampsia and normotensive controls. In addition, we found significant associations between third trimester SUA/sCr values and the development of preeclampsia and composite maternal and neonatal outcomes. Therefore, we suggest that SUA/sCr may have a predictive role for both preeclampsia and preeclampsia-associated complications and may help differentiating between women who will develop HDP-non-preeclampsia and those who will develop preeclampsia.

## ACKNOWLEDGEMENTS

### Conflicts of interest

There are no conflicts of interest.
